# Nutrient Acquisition and Attachment Strategies in Basal Lineages: A Tough Nut to Crack in the Evolutionary Puzzle of Apicomplexa

**DOI:** 10.3390/microorganisms9071430

**Published:** 2021-07-02

**Authors:** Andrea Valigurová, Isabelle Florent

**Affiliations:** 1 Department of Botany and Zoology, Faculty of Science, Masaryk University, Kotlářská 2, 611 37 Brno, Czech Republic; 2 Parasites and Free-Living Protists (UMR7245 CNRS-MNHN, MCAM), Department “Adaptations of living organisms”, National Museum of Natural History, CEDEX 05, 75231 Paris, France; isabelle.florent@mnhn.fr

**Keywords:** apical complex, attachment, epimerite, feeder organelle, mucron, myzocytosis, nutrition, parasitophorous vacuole/sac, pores, trophozoite

## Abstract

Apicomplexa are unicellular eukaryotes that parasitise a wide spectrum of invertebrates and vertebrates, including humans. In their hosts, they occupy a variety of niches, from extracellular cavities (intestine, coelom) to epicellular and intracellular locations, depending on the species and/or developmental stages. During their evolution, Apicomplexa thus developed an exceptionally wide range of unique features to reach these diversified parasitic niches and to survive there, at least long enough to ensure their own transmission or that of their progeny. This review summarises the current state of knowledge on the attachment/invasive and nutrient uptake strategies displayed by apicomplexan parasites, focusing on trophozoite stages of their so far poorly studied basal representatives, which mostly parasitise invertebrate hosts. We describe their most important morphofunctional features, and where applicable, discuss existing major similarities and/or differences in the corresponding mechanisms, incomparably better described at the molecular level in the more advanced Apicomplexa species, of medical and veterinary significance, which mainly occupy intracellular niches in vertebrate hosts.

## 1. Introduction

Apicomplexa Levine 1970, emend. Adl et al. 2005 inhabit almost all known phyla of metazoan organisms and are currently among the most monitored groups of unicellular eukaryotic parasites. Many are causative agents of human and animal diseases (including malaria, toxoplasmosis, babesiosis, coccidiosis and cryptosporidiosis) that are major world health problems and have a considerable impact on the global economy. Apicomplexans are parasitic protists whose invasive stages, termed zoites, are heteropolar cells possessing a so-called ‘apical complex’ comprising cytoskeletal and secretory organelles [1,2,3]. Recent transcriptomic studies indicate that apicomplexan parasites in the traditional sense are polyphyletic [4,5].

Apicomplexa are hypothesised to have evolved from photosynthetic (mixotrophic) free-living ancestors. This hypothesis is supported by the fact that free-living predatory flagellates (colpodelids) and photosynthetic coral symbionts (chromerids), combined in the clade Chrompodellida [6], are phylogenetically close (sister group) to Apicomplexa. Ancestral apicomplexan parasites, thought to have originally been restricted to marine annelids, most likely then spread to other groups of marine invertebrates, then to freshwater and terrestrial invertebrates, and finally to infect vertebrates [2]. It appears that parasitism in Apicomplexa arose independently at least three times [4,5,7]. Blastogregarines, archigregarines, eugregarines with neogregarines, agamococcidia, protococcidia and cryptosporidia, have been hypothesised as early branching apicomplexans [8,9,10,11,12,13,14,15,16,17], with some of them (agamococcidia, protococcidia) recently revealed as sister lineages to the medically important groups represented by Coccidia [4]. These basal groups exhibit enormous diversity in their cell dimensions and architecture (correlating with their surrounding environment and parasitism strategy) and are hence highly informative examples of coevolution between parasites and their hosts, the study of which could provide novel views on the acquisition of adaptive traits during the evolutionary history of Apicomplexa [18].

Under distinct evolutionary pressures, apicomplexan parasites have evolved a remarkably wide diversity of specific adaptations to reach and survive within hosts’ niches, leading to huge diversification of host–parasite interactions, perfectly reflected in their strategies for invasion/attachment to specific cell types and nutrient acquisition. Nevertheless, most of the knowledge acquired so far regarding these host–parasite interactions is highly biased towards a few emblematic species represented by intracellular vertebrate parasites and causative agents of major human and animal diseases (i.e., apicomplexans generally considered to be evolutionarily advanced) such as *Plasmodium* and *Toxoplasma*. In contrast, deeper branching apicomplexans that are mostly restricted to invertebrate hosts have remained almost unexplored for decades due to their limited practical significance. At present, however, greater importance is given to research on these basal lineages as they can contribute to our overall understanding of the evolution and biology of all Apicomplexa.

In recent years, several works focusing on selected representatives of basal apicomplexan lineages have been published, and new questions raised, gradually revealing some specific and original features displayed by these indeed unique organisms. The aim of this review is to summarise currently available information on the strategies displayed by the groups of basal apicomplexans to attach their vegetative stages to host cells/tissues and to take up food. These features, mainly described at morphological and ultrastructural levels, allow the tentative establishment of possible evolutionary trends that have led to the emergence of parasitic strategies developed by their more evolutionary advanced apicomplexan cousins infecting vertebrates, which are currently the subject of much more detailed investigations at molecular and functional levels.

## 2. Apical Complex Structure and Function

The invasion of vertebrate host cell(s) by apicomplexans is a rapid process that has been very well described for *Plasmodium* and *Toxoplasma* and depends on a sequence of strictly controlled events leading to the attachment of the parasite to and subsequent invasion of the host cell. To achieve this goal, apicomplexans have evolved highly specialised motile invasive stages called zoites, equipped with a cytoskeleton and a set of sophisticated organelles located at their anterior pole that are designed to reach and invade the host cell. By possessing this specific invasion apparatus, known as the apical complex, apicomplexan zoites exhibit high cell polarity. The apical complex of Apicomplexa is a highly organised and dynamic structure, composed of a cytoskeletal backbone forming an apical cone capable of protrusion (usually comprising a closed conoid (secondarily lost in some species), apical polar ring(s), spirally arranged subpellicular microtubules and a large collection of associated proteins) that scaffolds a group of extrusive vesicular membrane-bounded organelles (rhoptries, micronemes and dense bodies) known to be used sequentially for host-cell invasion by vertebrate apicomplexan parasites [19,20,21]. This apparatus is usually disassembled after the final localisation of the apicomplexan parasite in the host cell. The apical complex was first identified at the ultrastructural level, through extensive electron microscopy studies covering a wide range of species. Because of its key role in parasite invasion/attachment to the host cell, this apparatus has attracted the attention of biologists for decades as a promising target for therapeutic drug and vaccine interventions against vertebrate apicomplexan pathogens [19].

### 2.1. From Invading Apicomplexan Zoites to Vegetative Trophic Stages

Molecular explorations of apical complex architecture and functioning have mainly been conducted on *Plasmodium falciparum* and *Toxoplasma gondii*, thereby establishing a comprehensive framework on which comparative studies across Apicomplexa and beyond can now be undertaken [21,22]. Hundreds of proteins are currently known to be involved in the structure and function of apical complexes/invasion machineries, including proteins of the inner membrane complex (IMC); specific cytoskeletal elements; and protein contents of rhoptries, micronemes and dense granules [23]. It is now well established that host-cell invasion by vertebrate parasites (in the general scheme expected to be mostly applicable to intracellular apicomplexans) is a highly orchestrated multistep process enabled by parasite movement (gliding) and starting with (1) host-cell recognition and attachment of the parasite to the host cell, mediated by microneme-secreted adhesins; (2) parasite reorientation relative to the surface of invaded cell; (3) establishment of a parasite-encoded ‘moving-junction’ device formed by micronemal apical membrane antigen 1 (AMA-1) and several rhoptry neck proteins (RONs, such as RON2/4/5/8 in *Toxoplasma gondii*), anchoring the parasite to the host cell; and finally (4) rapid parasite entry by a gliding movement and concomitant creation of a parasitophorous vacuole (PV) that closes in a twist [21,24]. Finally, some rhoptry bulb proteins (ROPs) and dense granule proteins (GRAs) help establish the parasite–host cell interface at the level of the parasitophorous vacuole membrane, while additional ROPs and GRAs transit to host cell cytoplasm and even to the host cell nucleus, allowing a parasite-driven host-cell manipulation [25]. Overall, comparative studies across vertebrate apicomplexans have established that the structures and adhesive domains of micronemal proteins tend be well conserved while ROPs and GRAs appear to be highly genus- or even species-specific [25,26,27]. Variations of this scheme, however, are to be expected in apicomplexans with extracellular or epicellular trophozoites, such as invertebrate parasites (e.g., gregarines and blastogregarines), and some vertebrate apicomplexans. For example, in *Cryptosporidium* spp. and coccidian *Goussia janae* that both occupy epicellular positions in their respective vertebrate epithelial host cells and do not penetrate HCPM, the molecular elements of the ‘moving junction’ have either been lost (*Cryptosporidium* lacks both the AMA1 and RON2/4/5/8 orthologues) or kept (*Goussia*) [28,29]. In addition, even intracellular parasites such as *Theileria* invade their host cell (lymphocytes) by yet another mode, termed zippering [22].

The apical complex, however, is not only used for host-cell invasion as it has also been shown to be involved in host-cell attachment and feeding in some basal apicomplexans. Nevertheless, in contrast to medically significant vertebrate pathogens, although a large series of microscopic investigations has proven the involvement of the apical complex in this attachment/feeding process in apicomplexans from invertebrate hosts (e.g., blastogregarines, gregarines, protococcidia and agamococcidia), not one molecular participant has been identified so far. The biological questions are, however, numerous. There is a need to identify which of the molecular elements described as constituting the apical complex in vertebrate parasites (*Toxoplasma*, *Plasmodium*—or even *Theileria*, *Babesia*, *Eimeria*, *Cryptosporidium*) also compose the apical complex and/or which of them are missing in apicomplexan parasites of invertebrates. This knowledge could help to understand why vertebrate apicomplexans appear to prefer invading host cells to establishing intracellular niches, whereas most invertebrate parasites (blastogregarines, gregarines, protococcidia) do not, and whether the invertebrate parasites involve additional actors that explain their different functions. Are these additional actors also present in proto-apicomplexan *Chromera velia* and *Vitrella brassicaformis* or in other Myzozoa known to possess apical complex structures resembling those of Apicomplexa? To what extent are innovations in the edifice/proteins of the apical complex responsible for this particular evolution?

### 2.2. From Myzocytosis to Full Invasive Capacity

In contrast to the well-known intracellular apicomplexan parasites of vertebrates, epicellular trophozoites of plesiomorphic apicomplexans such as blastogregarines and archigregarines retain their apical complex for myzocytosis-based feeding [9,14,16,17,30]. Myzocytosis, a primarily predatory trophic mode, which is based on penetration of the prey surface and sucking of its contents via specialised organelles morphologically similar to those used by the apicomplexan zoites for invasion [31], is characteristic of Myzozoa—a group of very diverse unicellular organisms comprising apicomplexans, dinoflagellates and several different lineages of free-living predatory or parasitic flagellates [32,33]. A microtubular-based structure, called the conoid, is widely involved during myzocytosis [34,35]. Most Myzozoa are biflagellates, with the notable exception of all apicomplexans which have virtually lost these flagella (although one still remains in the male gametes of some species) [19,36]. It is now proven that in some intracellular parasites of vertebrates, former components of these flagella were reinvested in the formation of the apical complex, an emblematic structure of Apicomplexa directly linked to the conoid [19]. Based on the presence of a conoid, Apicomplexa are divided into Aconoidasida (comprising Haemospororida, Piroplasmorida and poorly studied Nephromycida), which possess an apical complex lacking the conoid in asexual motile stages, while some diploid motile zygotes (ookinetes) may have retained it (heteroxenous parasites) [1], and Conoidasida, which possess a complete apical complex with a closed conoid in all or most asexual motile stages. Conoidasida, at present considered as polyphyletic with several artificial and unclear subdivisions [1], comprise homoxenous and heteroxenous parasites including coccidia (Adeleorina and Eimeriorina), gregarines (Archigregarinorida, Eugregarinorida, Neogregarinorida), cryptosporidia and Blastogregarinea that have been shown to be an independent, early diverging lineage of Apicomplexa [17]. It was recently proposed that *Cryptosporidium* spp. be included in Gregarinasina as Cryptogregarinorida [1], but shortly thereafter the proposal was questioned [7] and later abandoned [37].

It has been hypothesised that the apical phagotrophy observed in alveolate free-living predators with an open conoid and rhoptries may be at the origin of the attachment and feeding apparatus of archigregarines [16,33]. As *Selenidium pendula* represents an early branching apicomplexan and an archigregarine type species, its conoid could be a reference model to study the transition between the free-living alveolate ancestors with an open conoid (like that found in the early branching dinoflagellates) and apicomplexans with a closed conoid [16,33,35]. Recent studies indicate that Apicomplexa display two main evolutionary trends: (i) the origination of epicellular parasitism restricted to the apical surface of the host cell, mostly observed in gregarines and cryptosporidia, with subsequent modifications to their attachment apparatus and motility mode at the trophozoite stage, and (ii) origination of intracellular parasitism, typical of Coccidia and Aconoidasida, accompanied by loss of polarity and motility in intracellular trophozoite stages following host-cell invasion by zoites [15]. It is possible that the evolution of Apicomplexa progressed from myzocytotic predation (ancestors) to myzocytotic extracellular parasitism (blastogregarines, gregarines, cryptosporidia), accompanied by the origination of epicellular parasitism, and finally to intracellular parasitism as observed in more evolutionarily advanced Apicomplexa. The idea that modifications of the apical complex allowed apicomplexan parasites to switch from an extracellular life mode in gregarines to an intracellular life mode in vertebrate parasites is not new [34], but the molecular actors and mechanisms underlying this transition are currently unknown [28]. It is fairly well documented that heterotrophic modes have evolved in eukaryotes from ancestral phagotrophy to derived osmotrophy [38,39,40]. Phagotrophy has apparently been lost in Apicomplexa while it has been retained in the two other alveolate lineages (ciliates and dinoflagellates), but it may be relevant to propose that, from a common phagotrophic ancestor, myzocytosis represents an intermediary trophic mode along the continuum towards osmotrophy, the main trophic mode of *P. falciparum* and *T. gondii,* for example. In that sense, exploring and comparing archigregarine and eugregarine genomes versus other apicomplexan osmotrophs may help establish whether remnants of the phagotrophic mode displayed by the myzozoan ancestor can be traced back in some early branching representatives.

The broad distribution of apical complexes with rhoptries, micronemes and pseudoconoids in free-living relatives of Apicomplexa (photosynthetic *Chromera*, predatory *Colpodella* and *Psammosa* and more distantly related parasitic genera *Perkinsus* and *Parvilucifera*) points to a single origin of the apical complex in an apicomplexan and dinoflagellate ancestor in a nonparasitic context [4]. Because of its secretory involvement in the formation of cell-to-cell interactions, it could be considered an important precondition for the multiple origins of parasitism in dinoflagellates and apicomplexans. The functioning of the apical complex in extracellular attachment and secretion in a similar host environment (host intestinal epithelium) may have triggered convergent expansion in the cell dimensions and led to convergent parasite morphologies [4]. Indeed, the most recent studies showed that large trophic stages attached to the gut of marine invertebrates by highly specialised apical devices are the products of convergent evolution, assumed to act in similar preconditions [4]. The morphological similarities, however, are usually only superficial, while considerable differences can be found at ultrastructural level. Examples of independent emergence of apicomplexan-like parasitism can be found in enigmatic organisms such as *Platyproteum* (originally described as an archigregarine) forming a new lineage basal to apicomplexans and chrompodellids and *Piridium sociabile* (previously classified as schizogregarine) clustering away from Apicomplexa and representing a sister taxon to *Vitrella* [5]. Another example is *Digyalum oweni*, a formally described archigregarine and recently shown to be a sister lineage to all apicomplexans and chrompodellids, that indeed bears a modified apical complex and pellicle that differs from those in gregarines [4]. The ‘mucron’ of *D. oweni* trophozoites attached to the rectal epithelium of a snail (*Littorina obtusata*) comprises a ring of lobes embedded in the host cell, filled with cytoplasm with infrequent fibrils and microtubules and covered by a single membrane, and an adjoining ring of dense granular material. Although lacking the conoid, this attachment structure contains an apical complex with subpellicular microtubules and a protruded polar ring, which provides a gateway for rhoptry-mediated secretion—a combination of traits typical of gregarine apicomplexans [4,41,42].

## 3. The Niche and Feeding Strategies in Vegetative Stages

The attachment apparatus of basal Apicomplexa, which evolved precisely at the apical end of the zoite, exhibits vast diversity in its architecture depending not only on the species of parasite but also on the host and host cell types. The structures or organelles that attach the parasite to the host tissue/cell in apicomplexans from different phylogenetic branches often demonstrate external similarity associated with a similar functional load. Indeed, a recent study on gregarine parasites raised the question of the need to classify attachment organelles based on their origin, organisation and participation in the transport of substances from the host to the parasite [43].

### 3.1. Epicellular Parasitism

Sporozoites of most eugregarines, archigregarines and blastogregarines develop into large extracellular heteropolar vegetative stages called trophozoites, whose apical end, equipped with specialised organelles, enables them to remain attached to the luminal (apical) surface of host epithelial cell. However, although the sporozoites of these organisms show a subcellular organisation characteristic of apicomplexan zoites, their fate varies depending on the subsequent modifications of their apical end during the invasion/attachment to the host cell. Blastogregarines and gregarines indeed display a huge range of host–parasite interactive strategies depending on the type of attachment device: (i) intracellular or intratissular localisation, with or without a reduced attachment region in neogregarines; (ii) mucron in blastogregarines [17,44], archigregarines [14,16,30,45,46], some neogregarines and similar organelles in monocystid eugregarines considered to be a mucron by some authors [47] (although reliable TEM data are lacking); (iii) a more advanced mucron-like structure in aseptate (trophozoite composed of a single cell compartment bearing the attachment organelle) eugregarines that has lost the apical complex and enhanced its attachment function [48,49]; (iv) a simple epimerite, or (v) a complicated epimerite equipped with diverse structures (e.g., digitations, hairs, hooks, spines) in most eugregarines [11,12,47,50,51,52]; and finally (vi) a modified protomerite with rhizoids or sucker-like protomerite in few septate (trophozoites subdivided into a protomerite bearing the attachment device and a deutomerite with a nucleus) eugregarines [12,53].

**Figure 1:**
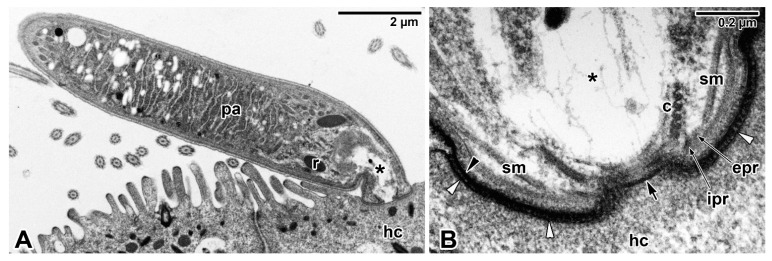
Epicellular parasitism in blastogregarine *Siedleckia nematoides* from the intestine of a polychaete *Scoloplos* cf. *armiger*. (**A**) General view of an attached trophozoite with a well-developed mucron. (**B**) Detailed view of a parasite apical region and the host–parasite interface (TEM). *asterisk*—mucronal vacuole, *black arrow*—outlet opening of the mucronal vacuole, *black arrowhead*—parasite plasma membrane, *c*—conoid, *epr* and *ipr*—external and internal parts of the polar ring, *hc*—host cell, *pa*—parasite, *r*—rhoptry, *sm*—subpellicular microtubules, *white arrowhead*—host cell plasma membrane. All micrographs used in this review are from the archives of A. Valigurová and none of them have been published elsewhere.

**Figure 2:**
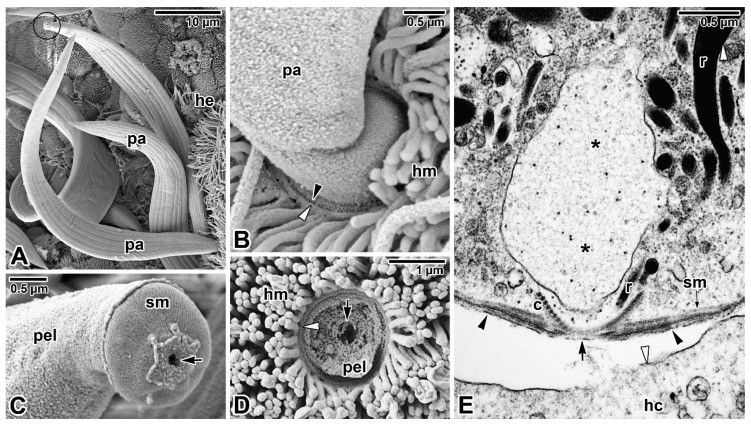
Epicellular parasitism in archigregarine *Selenidium hollandei* from the intestine of polychaete *Sabellaria alveolata*. (**A**) General view of trophozoites attached to the host intestine and one detached parasite revealing its former attachment site (encircled) (SEM). (**B**) Detail of the apical region of the parasite embedded in the brush border of host intestinal epithelium (SEM). (**C**) Detailed view of apical end of a detached parasite showing its mucron area with a central opening (the pellicle covering this region is peeled off, revealing the layer of subpellicular microtubules) (SEM). (**D**) View of the brush border of the host intestine with remnants of a parasite broken (presumably during sample processing) at its apical end, revealing the parasite mucron embedded within the host microvilli/microcilia and a central hole corresponding to the outlet of the mucronal vacuole (SEM). (**E**) View showing the apical region of a freshly detached parasite with a mucronal vacuole and an opening through which myzocytosis occurred, as well as the obviously damaged plasma membrane of the host cell (TEM). *asterisk*—mucronal vacuole, *black arrow*—outlet opening of the mucronal vacuole, *black arrowhead*—parasite plasma membrane, *c*—conoid, *hc*—host cell, *he*—host epithelium, *hm*—host cell microvilli, *pa*—parasite, *pel*—parasite trilayered pellicle, *r*—rhoptry, *sm*—subpellicular microtubules, *white arrowhead*—host cell plasma membrane.

Although the feeding strategies used by gregarines have been the subject of extensive debate, the exact mechanisms for nutrient uptake from their hosts and/or their environment remain unclear. The most recent studies focusing on gregarine groups from various environments indicate that their feeding modes largely depend on the long-term environmental conditions forming their specific niche. Correlations between the environment occupied within the host body and the characteristics of gregarine trophozoites are often discussed [45]. It has been shown that the most plesiomorphic apicomplexans, archigregarines (considered to be the earliest diverging lineage of Apicomplexa) and blastogregarines, have retained myzocytosis as their principal mode of feeding [17]. However, one relatively fundamental difference can be found here; while archigregarines have nonfeeding gamonts (but their conoid and rhoptries persist until at least progamic mitosis starts), in blastogregarines, the mucronal complex remains active and performs myzocytosis throughout the lifespan of the attached parasite [17,30,47]. Both these apicomplexan parasites of marine polychaetes attach to the host enterocyte’s brush border that bears the microvilli and microcilia, via an attachment structure called the mucron (Figure 1A,B, Figure 2A–E and Figure 9A). Typically, the trimembrane pellicle, formed by the parasite plasma membrane (PPM) underlain by the IMC, covers the mucron, except for the small region against the conoid where a vacuole called the mucronal vacuole has a wide inlet opening (Figure 1B and Figure 2D,E). This structure, considered as a temporary cytostome–cytopharyngeal complex (= cytostome), performs the myzocytosis.

While many authors agree that this feeding mode is made possible by the preservation of organelles of the apical complex in the trophozoite mucron, only a few ultrastructural investigations succeeded in revealing the process of myzocytosis or at least its indications. The process of myzocytosis is well illustrated in *Selenidium* archigregarines [16,30]. Their mucrons usually have the appearance of a regular mammiliform area with a series of shortened microvilli at its periphery when embedded in the host epithelium. The regular trace left by the mucron of a detached trophozoite in the host epithelium sometimes contains a small subcentral hole [16]. Detailed ultrastructural observations revealed the mucronal vacuole to be inserted into the conoid and surrounded by abundant micronemes and rhoptries (Figure 2E). 

The myzocytosis, starting at the conoid apex, and the continuity of the membrane limiting the mucronal vacuole up to contact with the host epithelial cell strongly suggest that the parasite indeed sucks the nutrients from the host cell using this parasitic evagination through the conoid apex, followed by fragmentation of the initial mucronal vacuole into numerous smaller vacuoles, which are transported via a microtubular network into the parasite body [16,17,30]. Abundant small vesicles (most likely pinocytotic) found at the periphery of these vacuoles resemble the micropinocytic vacuoles observed in vorticellid ciliates [47]. Subsequently, the disrupted site of the host cell plasma membrane (HCPM) (Figure 2E, local lysis due to secretion of parasite rhoptries) is restored [30]. Some studies reported the existence of an axial streak of optically distinct cytoplasm extending from the anterior to the posterior end in *Selenidium* archigregarines, forming an expansion around their nucleus and likely representing a nutrient distribution system [14,54]. The presumably digestive vacuoles detected in this axial row appear to correspond to the above-mentioned fragmented mucronal vacuole involved in myzocytosis and may transport nutrients posteriorly along the longitudinal cell axis. The number of vacuoles surrounding the nucleus in *S. pendula* supports this hypothesis [55]. Whether these vacuoles are trafficked towards a lysosomal-like organelle where molecular digestion is achieved remains to be further investigated. The localisation of acid phosphatase, reported to be restricted to the rhoptries of *S. hollandei* trophozoites, rather corresponds to the mucronal vacuole or fragments migrating posteriorly into the archigregarine body, which, together with the detection of this enzyme in the apical vacuoles of host enterocytes, suggest a nutritional function and an active process occurring between the parasite mucronal complex and the host cell [56,57]. Accordingly, this enzyme was detected in the ‘electron-lucent vacuoles’ of *S. cometomorpha* and *S. virgula*, while no acid phosphatase was associated with their rhoptries [57,58]. A tropism for host cells rich in granules has been observed in intestinal epithelium parasitised by *S. pendula* [16], suggesting either parasite tropism for nutrient-enriched cells or a capacity for the parasite to secondarily attract host cell granules in the proximity of its attachment area.

Some differences have been identified among the mucronal complexes of *Selenidium* archigregarines and blastogregarines studied so far. For example, the conoids of *S. pendula*, *S. hollandei* and *S. orientale* closely resemble those of *T. gondii* and eugregarine sporozoites, but they appear to lack the apical polar ring [16,30,56,59,60] that represents a microtubule-organising centre unique to Apicomplexa. Instead, an IMC dilatation located in the corresponding region appears to function in generating the subpellicular microtubules in these archigregarines, in contrast to *S. pherusae*, in which the polar ring, located adjacent to the IMC at the conoid apex, gives rise to subpellicular microtubules [14]. Similarly, in two blastogregarine species so far investigated using electron microscopy, ultrastructural differences have also been reported at the mucron level. While the mucronal complex of *Siedleckia nematoides* is well developed and equipped with the mucronal vacuole and a typical set of apical organelles comprising the conoid, polar ring (subdivided into internal and external parts of different electron density), numerous rhoptries and putative micronemes (Figure 1A,B), the strongly modified mucron of *Chattonaria mesnili* lacks both the conoid and rhoptries and anchors to the host cell via peripheral bulges formed by large alveoli between the cortical cytomembranes of the IMC [17,44]. While the *S. nematoides* cytostome opens into a tiny gap between the HCPM and PPM, with the appearance of a septate cell junction, in *C. mesnili,* the gap is of varying width between the parasite and host cell and lacks any evidence of a septate cell junction. It is not clear whether these ultrastructural differences in the host–parasite interface, where nutrient exchange likely occurs, result in distinct modes of feeding in these two blastogregarines. The host epithelial cells parasitised by blastogregarines show no significant modifications, only the HCPM directly facing the parasite cytostome has increased electron density and its external surface bears uniformly spaced dense structures (presumably a perforated or modified host cell coat) [17]. 

**Figure 3:**
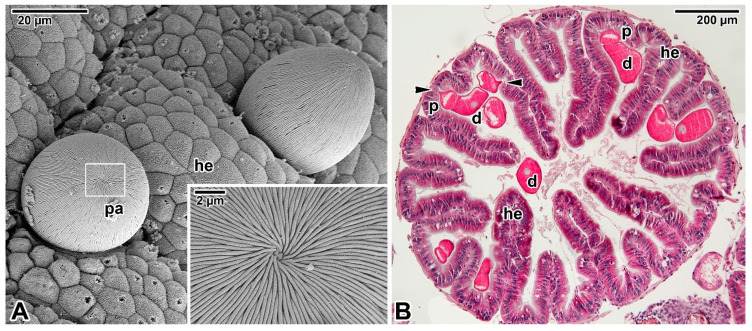
Epicellular parasitism in aseptate (**A**) and septate (**B**) eugregarines. (**A**) Trophozoites of *Lecudina tuzetae* attached to the intestine of polychaete *Hediste* (*Nereis*) *diversicolor*; the insert shows a detail of the longitudinal epicytic folds covering the eugregarine posterior pole marked by a white rectangle (SEM). (**B**) Various stages of trophozoites of *Gregarina garnhami* in the caecum of a desert locust *Schistocerca gregaria* (LM—histological section stained with haematoxylin and Best’s carmine). *black arrowhead*—epimerite, *d*—deutomerite with a single nucleus, *he*—host epithelium, *p*—protomerite, *pa*—parasite.

Far greater ambiguities remain concerning the modes of nutrition displayed by eugregarines (Figure 3A,B) as they do not appear to feed through the myzocytosis, except perhaps in their earliest developmental stages, i.e., when the sporozoite is transforming into a trophozoite stage in which the apical complex is gradually reduced and replaced by a complicated attachment apparatus such as epimerite or mucron-like organelle [15,61]. Most authors tend to attribute a feeding function in eugregarines to these attachment structures [62]. The architecture of the epimerites varies significantly between eugregarine genera, appearing to mainly depend on the host diet. It is remarkable that while epimerites in herbivorous hosts are usually simple and button-shaped, in carnivorous hosts they are much more complicated, being equipped with strong hooks, spines or filaments [62]. The shape of the epimerite also depends on the density of the surrounding environment; in a denser (hypertonic) environment, the epimerite protrusions become more pronounced, while in a hypotonic environment they shrink and disappear [63]. 

Despite differences in their final appearance, epimerites in different species generally share certain key characteristics, particularly the fact they are covered only by the PPM with an underlying cortical vesicle (in contrast to the rest of gregarine covered by the trimembrane pellicle comprising the PPM and IMC) and that the precursor of the epimerite is an epimeritic bud arising above the opened apical region of the invading sporozoite [11,12,50,52,59,64]. Electron microscopic monitoring of the individual steps that occur sequentially during the invasion process and sporozoite transformation into the trophozoite revealed stage-specific structures in all eugregarines studied. These included the transient presence of a flask-like organelle with an opening towards the apical pole and passing through a conoid in the earliest trophozoites [11,12,50,60,64]. This organelle, which is electron-dense in the youngest parasites, turns electron-lucent when the epimeritic bud and consequently the cortical vesicle is formed, suggesting it corresponds to a rhoptry emptying its contents. Another possible explanation is that the flask-shaped organelle is in fact a digestive vacuole that acts during myzocytosis in the earliest developmental stages attached to the host epithelium and corresponds to the mucronal vacuole documented in archigregarine and blastogregarine hypersporozoites [16,17,65]. This could be the case, for example, for the sporozoites of *Ascogregarina* (*Lankesteria*) *culicis* attached to the *Aedes aegypti* enterocytes [60]. The presence of the flask-shaped organelle in earliest stages of this eugregarine, which passes through the conoid and contains heterogeneous material (resembling pieces of host cell organelles and debris) at different stages of digestion, is indicative of a phagocytic process along with active digestion within this membrane-bound structure.

**Figure 4:**
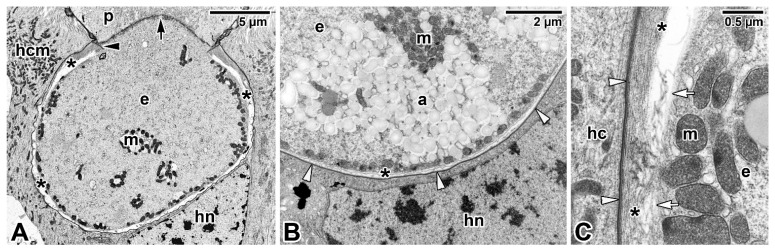
Epimerite of eugregarine *Gregarina garnhami* from the intestine of a desert locust (*Schistocerca gregaria*). (**A**) General view of the well-developed epimerite in a maturing trophozoite. (**B**) The apical region of epimerite packed with abundant amylopectin granules typical of mature trophozoites. (**C**) Detailed view of the interface between the epimerite and the host cell (TEM). *a*—amylopectin granules, *asterisk*—cortical vesicle, *black arrow*—epimeritic septum, *black arrowhead*—osmiophilic ring (= fusion site of the parasite and host cell plasma membranes), *e*—epimerite, *hc*—host cell, *hcm*—host cell mitochondria, *hn*—host cell nucleus, *m*—parasite mitochondria, *p*—protomerite, *white arrow*—membranous structure limiting the cortical vesicle, *white arrowhead*—host–parasite interface.

**Figure 5:**
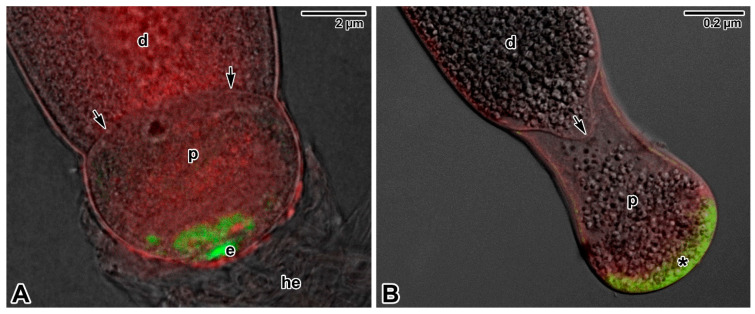
Tubulin-rich (green) structures in eugregarine attachment organelles. (**A**) Tubulin-rich ring at the interface between the protomerite and minute epimerite of *Cephaloidophora* cf. *communis* from the intestine of a barnacle (*Balanus balanus*). (**B**) Modified protomerite of *Gregarina cuneata* from the intestine of a mealworm larva (CLSM in a combination with transmission LM—composite views, IFA-FITC/phalloidin-TRITC). *asterisk*—apical protomerite region, *black arrow*—epimeritic septum, *d*—deutomerite, *e*—epimerite, *he*—host epithelium, *p*—protomerite.

The cortical vesicle is thought to form by the fusion of flat vesicles, first distributed at the epimerite periphery and most likely originating from the endoplasmic reticulum, which then turn into a single large vesicle packed with microfilaments [64]. The varying degrees of reduction in the size of the cortical vesicles in some epimerites is likely related to their convoluted character, which significantly increases their absorptive surface [12,61]. In *Didymophyes gigantea,* the cortical vesicle has been interpreted as the periparasitic space between the host cell and the parasite, functioning as a PV [61]. The electron-translucent cortical vesicle with traces of opaque and/or filamentous material, and in some eugregarines with additional fine tubular structures passing through the cortical vesicle and attaching to the epimerite–host cell interface, indeed resembles the internal space of the PV [11,51,66]. However, the true interface between the eugregarine and the host cell lies above the cortical vesicle and is trilaminar, consisting of HCPM and PPM covering the epimerite separated by a dense layer. Thus, the cortical vesicle is hypothesised to be an incomplete PV limited to the apical region of eugregarines (= epimerites) embedded in the host tissue and part of the parasite itself [15]. This hypothesis is supported by observations of subsiding and irregular appearance of the cortical vesicle during the gradual regression of the epimerite before detachment of the trophozoite from the host epithelial cell [52]. The maturing trophozoite remains epicellular, and the only part that remains in close contact with the host cell is the PPM covering the epimerite (Figure 3A,B and Figure 4A). The growing epimerite is gradually implanted in the host cell, causing deep invagination of the HCPM but not its penetration [11,12,52,64,67]. An osmiophilic ring (= membrane fusion site) is formed, interconnecting the HCPM, the epimerite PPM and the membrane limiting the cytoplasmic face of the cortical vesicle (Figure 4A). Accumulation of actin at the epimerite base suggests that this ring is contractile [12,52,64,68,69]. In addition, in some species, the epimerite is separated from the protomerite by a fibrillar septum (Figure 4A) [51], which may even be supported by an α-tubulin-rich ring (Figure 5A) [48].

The abundance of endoplasmic reticulum in the growing epimerite of young trophozoites suggests activation of metabolic pathways [12] or creation of additional membranes. The feeding function of the epimerite is also supported by frequent observations of host cell mitochondria and endoplasmic reticulum concentrated around the eugregarine epimerite, accompanied by the location of parasite mitochondria just beneath the epimeritic cortical vesicle (Figure 4A–C), pointing to the existence of an active interaction between the epimerite and a parasitised cell and further supporting the hypothesis the epimerite is a metabolically active organelle [61,66,70,71]. Because epithelial cells with anchored epimerites of trophozoites usually show no apparent pathological changes, the cortical vesicle and vacuoles in the epimerite most likely absorb nutrients from the host cell through a membrane permeability-based mechanism (osmotrophy or pinocytosis), while abundant parasite mitochondria beneath the cortical vesicle could provide the energy required for nutrient absorption [51,52,62,64]. A similar mechanism is likely to ensure food intake in monocystid eugregarine *Nematocystis* through the extensively folded trilaminar contact zone between its mucron-like attachment device and the host cell. This folding considerably increases the contact zone between apposing HCPM and PPM, and tracer analysis using radioisotopes [D-glucose-6-^3^H] revealed a direct transition of metabolites from the host cell to the trophozoite by crossing this attachment site, while glucose is known to be a precursor of the reserve polysaccharide (=amylopectin) reported in various apicomplexans [49]. Other works on eugregarines (*Stylocephalus conoides*, *S. mesomorphi*, *Gregarina cuneata*, *Hirmocystis speculitermis*, *Nematocystis magna*, *Didymophyes minuta*) documented acid phosphatase, alkaline phosphatase and β-glucuronidase activities in the pellicle and cytoplasm of their trophic vegetative stages [57,72,73,74]. In addition, lipids, cholesterol and the activity of the key enzymes involved in the biosynthesis of steroids have been revealed in *S. conoides* trophozoites [75]. In gregarine-like non-apicomplexan parasite *D. oweni*, the rhoptries persist in both the young and mature trophozoites, and it has been suggested that in attached *D. oweni* parasites, the rhoptries secrete enzymes into the host cell prior to digestion. Although the host epithelium has been shown to be rich in esterases, acid phosphatase and alkaline phosphatase, the study only succeeded in visualising nonspecific esterases in the parasite cytoplasm [57]. However, if *D. oweni* indeed feeds by scavenging nutrients from the host cell and ingesting the host enzymes, then the apparent lack of acid phosphatase and alkaline phosphatase is more likely due to improper sample processing [41,57].

In species with a simple epimerite, two contradictory hypotheses have been proposed to elucidate the detachment of mature trophozoite from the host epithelium. While one describes self-regulated detachment of the parasite through retraction of the epimerite into the protomerite [51,52,66], the other refers to gradual constriction of the epimerite base by the contractile osmiophilic ring (which is thought to act as a sphincter) resulting in the complete separation of the epimerite from the rest of the gregarine body [47,62,64,68,76]. A similar mechanism could also be at work during the release of mature trophozoites attached to the host tissue by mucron-like organelles. The attachment organelle retraction-based trophozoite detachment seems more likely because the trophozoite development in eugregarines usually lasts more than four days [50,77]. Therefore, eugregarine trophozoites must either be adapted to keep the host cell alive until their full maturation or be able to abandon the senescing host cell and reattach to a younger epithelial cell in better physiological condition; the latter would be facilitated by a retractable epimerite and progressive gliding motility characteristic of eugregarines [12,52,66]. This is especially true in species equipped with permanent epimerites reminiscent of a modified protomerite that persist in sexual (gamont) stages (Figure 5A) [48]. The fact that the feeding stages of *G. cuneata* are able to separate from the host tissue and to retain an intact epimerite despite their deep anchorage in the host epithelium by numerous digitations [12] further supports the epimerite retraction hypothesis. This eugregarine, however, shows even more advanced adaptations to epicellular parasitism, namely the ability to take nutrients from the host intestinal epithelium in later developmental stages via the protomerite modified in its apical region. After epimerite retraction, early syzygies (pairing of two mature trophozoites/gamonts before the formation of a gametocyst and subsequent fertilisation) are often seen to be attached to the brush border of the intestinal epithelium via an undulated protomerite apex of the primite (anterior gamont in the syzygy). Increased accumulation of actin and tubulin (Figure 5B) in the protomerite apex may lead to increased flexibility of this region, thus enabling the parasite attachment [12,52,78,79]. Actinocephalid gamonts show a similar strategy for continuous feeding on the host epithelium; after the loss of simple globular epimerite in mature trophozoites, they reattach via a sucker-like protomerite [53]. In both these parasites with feeding gamonts, the interspace between the intestinal epithelium and the epicytic folds covering the attached protomerite region is filled with host microvilli deposited in dense adhesive material, most likely produced by the exocytic vesicles frequently observed in the apical cytoplasm of the parasite protomerite [12,53]. The frequent pores and ducts that regularly interrupt the apical pellicle on the *G. cuneata* protomerite, along with abundant vesicles, dense bodies and Golgi apparatus occupying the protomerite cytoplasm, are thought to be involved in nutrition/attachment via modified protomerite at the gamont stage. Similar pores with channels in the proximity of dense bodies (putative micronemes) documented in the mucron-like organelle of *Lecudina tuzetae* have been suggested to be involved in the secretion of parasite lytic enzymes [47]. In eugregarines from myriapods (e.g., *Grebnickiella gracilis*, *Echinomera lithobii*, *Cnemidospora lutea*, *Amphoroides circi*) the modified apical part of their protomerites forms a cup bearing numerous filaments (rhizoids) deeply inserted into the host enterocyte. These protomeritic expansions are covered only by the PPM, as seen in epimerites [47]. 

The feeding strategies may, however, differ between distant eugregarine taxa [12]. In lecudinids, a presumed local lytic effect on the parasitised tissue indicates a nutritional function of their mucron-like organelle through extracellular secretion of enzymes with subsequent absorption of externally predigested host tissue [62]. Increased accumulation of actin-like proteins in the mucron-like organelle of *Lecudina pellucida*, by its organisation corresponding to abundant 7 nm filaments documented by electron microscopy, supports its putative sucker function. Two possible mechanisms have been proposed to mediate the adhesion of mucron-like attachment devices (described at ultrastructural level in eugregarines belonging to the genera *Lecudina* and *Ascogregarina*) to the host cell: (i) contraction of the actin-like filaments [68,69] or (ii) successive ‘hydraulic’ evaginations and retractions of the mucron-like apex (= the thinnest part of the gregarine epicyte) due to contractions of the parasite body resulting in a suction process [80]. Interestingly, the large vacuolar areas forming an extensive branched system in the anterior part of the *A. culicis* and *A. barretti* trophozoites resemble the mucronal vacuole observed in true mucrons of archigregarines and blastogregarines [80,81]. Absorption and/or digestive functions have been proposed for this vacuolar system because the accumulation of electron-dense granular materials has been detected in its distal branches that deeply penetrate the whole length of the gregarine, suggesting it plays a role in the transport of nutrients absorbed through the PPM covering the mucron-like organelle [80]. Hence, similar attachment mechanisms could be expected in mucron (archigregarines, blastogregarines) and mucron-like organelles (eugregarines). Indeed, significant pathological effect on host epithelium demonstrated as drastic lytic modifications of host enterocytes in the form of a truncated cone showing a gradient of effects, likely induced by the parasite lysosomal enzymes along with the infiltration of dense material from the parasite mucron/mucron-like organelle into the host cell, has been reported in archigregarine *S. hollandei* and in eugregarine *Lecudina* spp. both from polychaete hosts [47]. The molecular makeup found in the different vacuoles described above (mucronal vacuoles, the epimeritic cortical vesicle, etc.) obviously built at the host–parasite interface of these basal apicomplexan representatives will need to be established (for example by subcellular proteomics) and compared to known situations to determine whether they correspond to known structures, such as digestive vacuoles or PV, or possibly represent an intermediate or mixed situation. Indeed, intracellular parasites of vertebrates do reside within their host cells surrounded by PV, which isolates the parasite from the host-cell defence and contains a wide variety of proteins of parasitic origin, allowing metabolic exchanges. The genomic exploration of gregarines is, however, still in its infancy [18]. 

**Figure 6:**
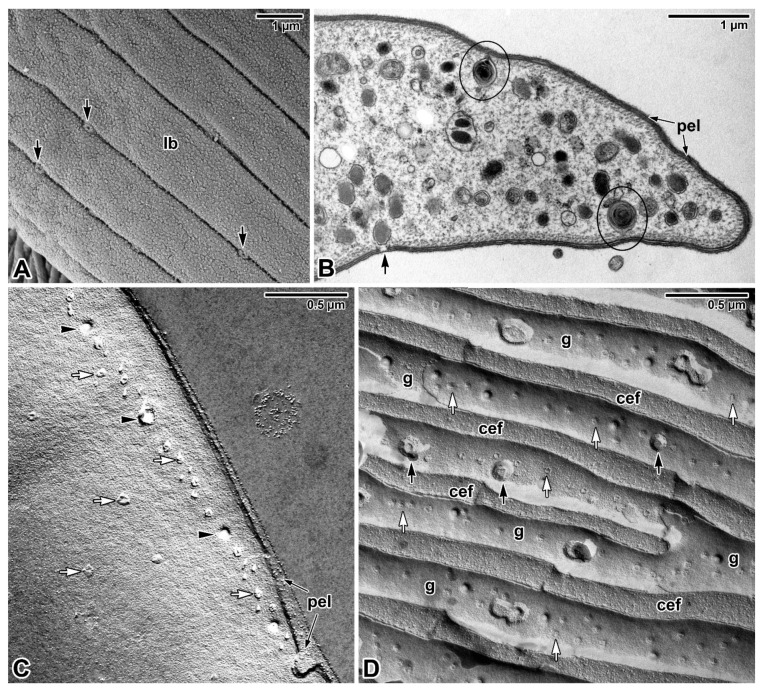
Distribution of pores on the cell surface of blastogregarines (**C**) and gregarines (**A**,**B**,**D**). (**A**) Pores arranged in rows in the grooves separating the epicytic longitudinal bulges (SEM) and (**B**) pinocytotic whorled vesicles (encircled) connected to openings (pores) obviously interrupting the pellicle covering the archigregarine *Selenidium hollandei* (TEM). (**C**) Freeze-fractured pellicle of blastogregarine *Siedleckia nematoides* revealing the abundant pores organised in a laterally located longitudinal row (FE TEM). (**D**) Freeze-fractured pellicle of eugregarine *Gregarina garnhami* with typical apicomplexan micropores and numerous smaller pores located in the grooves between the epicytic folds (FE TEM). *black arrow*—micropore, *black arrowhead*—large pores, *cef*—cytoplasm of epicytic folds, *g*—cytoplasmic face of the grooves between epicytic folds, *lb*—longitudinal bulges, *pel*—pellicle, *white arrow*—small pores.

That blastogregarines and gregarines also use alternative or additional modes of feeding to complete their life cycle is likely but remains to be explored. Given that gregarines generally grow even after separation from host tissue, one can speculate about other nutritional mechanisms, which is particularly true in the case of eugregarines (e.g., *Urospora* spp., *Lithocystis* spp., *Gonospora varia*, *Diplauxis hatti*) which develop in coelomic fluid without anchoring to the host tissue [12,13,47,62]. The growth of *G. blaberae* continues after trophozoite detachment, suggesting cell surface nutrition in stages devoid of their epimerites [82]. Alternative feeding modes could be (i) osmotrophy using a variety of dedicated transporters that are well described in *Toxoplasma*, *Plasmodium* or *Cryptosporidium* [83] or (ii) endocytosis—well documented at both the ultrastructural and molecular levels not only for *Plasmodium* erythrocytic stages (used to uptake and digest host-cell haemoglobin) but also for both the intracellular and extracellular stages of *T. gondii* [84]. Experimental assays with radioactive precursors performed on several eugregarine species (including the incubation of *G. garnhami* in [^3^S]cysteine or [^35^S]methionine; *Cephaloidophora conformis* in [^14^C]glucose, [^14^C]galactose and [^14^C]amidon; and *L. tuzetae* in [^3^H]uridine, [^3^H]leucine and [^14^C]glucose-1-phosphate) revealed the labelling of sites in trophozoites that could result from permeation pathways in the cortical cytomembranes [47]. The extensive pellicle folding (Figure 3A) in large eugregarine trophozoites appears to optimise surface-mediated nutrition (osmotrophy using a variety of transporters or pinocytosis via micropores/pores (Figure 6A–D) that need to be characterised both at functional and molecular levels) and may also explain the evolutionary loss of the apical complex with myzocytotic function in eugregarines, accompanied by the development of bulky attachment devices [32]. Endocytosis via micropores has been described in extraintestinal aseptate eugregarines—monocystid *Apolocystis* and urosporid *Cystobia* [47]. Likewise, the surface of *D. hatti* gamonts associated in syzygy covered by numerous hair-like microvilli (likely lacking the micropores) may enable the efficient osmotrophic nutrition required for the rapid growth of the syzygy, especially during the somatic transformation (epitoky) of their polychaete host (*Perinereis cultrifera*) [85]. Thus, questions arise as to whether and under what circumstances eugregarine attachment organelles serve for nutrient acquisition and whether the micropores are involved in pinocytosis [86] or serve to secrete the mucus involved in gliding motility, as proposed in other studies [79,87,88]. The presence of a single micropore located in the anterior third of the sporozoite in eugregarines *S. africanus* and *A. culicis* also suggests a possible role for micropores during feeding [59,60]. In advanced developmental stages of eugregarines, abundant micropores are found in deep grooves separating the epicytic folds (Figure 6D) with smaller pores randomly distributed on the lateral side or at the base of these folds [13,48,79,89,90,91]. Just to give an idea, it has been found that 8000–12,000 pores exist at the surface of the approximately 140 μm long trophozoite of *Rhynchocystis pilosa* [47]. 

As for representatives of basal lineages with hypertrophic zoites feeding via myzocytosis, there is ultrastructural evidence for pinocytosis in *Selenidium* archigregarines by means of pinocytotic whorled vesicles connected to pores and/or micropores arranged in rows and interrupting the pellicle covering the grooves between longitudinal bulges (Figure 6A,B) [14,16,92,93]. Interestingly, no typical micropores have been found in the blastogregarines studied, but in *S. nematoides*, vesicular structures containing a lamellar structure similar to that of *Selenidium* spp. are connected to the large pores that interrupt the IMC but not PPM [17,44]. Although the pellicle of *S. nematoides* bears numerous pores of three different sizes, predominantly organised in four laterally located longitudinal rows, none of these pores appear to pierce the PPM (Figure 6C) [44]. 

### 3.2. Epicellular Parasitism in the Embrace of the Host Cell Membrane

**Figure 7:**
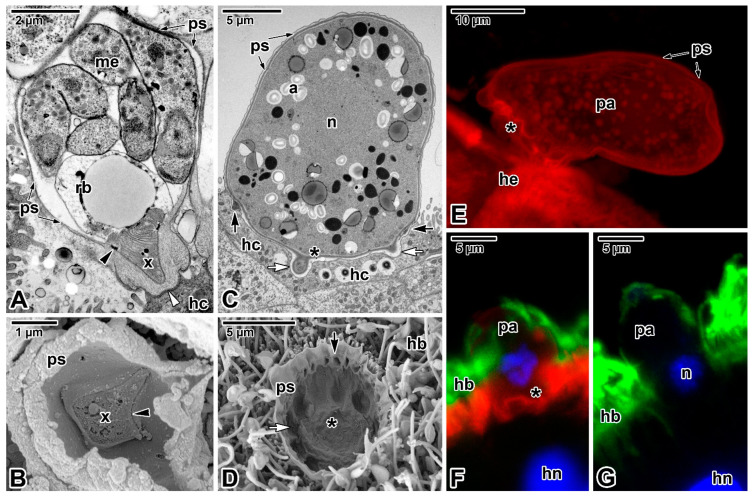
Epicellular parasitism in cryptosporidia (**A**,**B**) and protococcidia (**C**–**G**) encapsulated by a host cell apical membrane. (**A**,**B**) *Cryptosporidium muris* from the stomach of multimammate rats (*Mastomys natalensis*)—(**A**) a meront with merozoites (TEM); (**B**) an empty parasitophorous sac with a residuum of the feeder organelle (SEM). (**C**–**G**) *Eleutheroschizon duboscqi* from intestine of a polychaete *Scoloplos* cf. *armiger*—(**C**) a macrogamont (TEM); (**D**) a crater left after detachment of a trophozoite (SEM); (**E**) F-actin labelling (red) in a macrogamont covered by a parasitophorous sac (CLSM—composite view, phalloidin-TRITC); (**F**) colocalisation of α-tubulin (green) and F-actin (red) in a young trophozoite with a single nucleus (blue) showing the host microcilia adhering to the parasitophorous sac (CLSM—composite view, IFA-FITC/phalloidin-TRITC/DAPI); (**G**) α-tubulin labelling (green) in maturing trophozoite with a single nucleus (blue) revealing the presence of tubulin in the wall of the parasitophorous sac (CLSM—composite view of several median optical sections, IFA-FITC/DAPI). *a*—amylopectin, *asterisk*—attachment apparatus consisting of lobes and filamentous fascicles arranged in circles, *black arrow*—filamentous fascicles, *black arrowhead*—Y-shaped membrane junction, *hb*—brush border of the host intestinal epithelium, *hc*—host cell, *he*—host intestinal epithelium, *me*—merozoite, *n*—parasite nucleus, *pa*—parasite, *ps*—parasitophorous sac, *rb*—residual body, *x*—feeder organelle, *white arrows*—attachment lobes, *white arrowhead*—dense band separating the host cell into the modified part (=PS) and the unmodified part below it.

Unlike the epicellular gregarines and blastogregarines described above, which are not surrounded by host membrane, cryptosporidia (Figure 7A,B), protococcidian *Eleutheroschizon* (Figure 7C–G) and the archigregarine *Ditrypanocystis* all exhibit an extraordinary localisation within a host-derived parasitophorous envelope at the brush border of the host gastrointestinal epithelium ([11,15,94,95,96]; see explanatory schemes in [11,15,94,96]).

Opinions differ on the localisation of cryptosporidia developing in a peculiar niche within the host tissue; while some refer to them as intracellular extracytoplasmic parasites, others prefer the term epicellular to more accurately reflect their unique location in a host-derived parasitophorous sac (PS) [9,94,97,98,99,100]. Careful in vivo and in vitro observations of several species of gastric and intestinal cryptosporidia support the term epicellular as their invasive stages neither penetrate under the HCPM nor come into close contact with the host cell cytoplasm [11,94,95,101,102,103]. The invading parasite induces an actin-dependent modification of HCPM that loses its microvillous character and forms a circular fold gradually encapsulating the parasite [11,94,103,104,105]. The reorganisation of host actin and its accumulation at the parasite attachment site gives rise to a dense band formed by microfibrils interwoven perpendicularly with an adjacent filamentous network of polymerised actin, which separates the modified and unmodified host cell compartments, helps anchor the parasite and probably prevents it from penetrating the host cell cytoplasm [15,106,107,108,109]. As a result, the invasive stage remains epicellularly attached to the apical surface of the host cell, enveloped by the fold of the modified host cell membrane (Figure 7A). Simulated parasitisation of HCT-8 and HT-29 cell lines using polybeads coated with a ‘cocktail’ of parasite antigens induced the reorganisation of actin in the contacted cells, leading to the formation of an actin network surrounding the polybeads and their encapsulation by folds of the cultured cell membrane. This indicates that the encapsulation of cryptosporidia can be induced by parasite antigens and is thus independent of any active invasion by motile stages [95]. 

Cryptosporidia have been shown to possess unique features characterising their metabolism and biochemistry [110], but the exact mechanism of nutrient uptake is not yet clear. While the PS appears to play a protective role, the feeder organelle, representing the parasite attachment site and located at the base of PS (Figure 7A,B), could be the site that regulates nutrient and drug transport into the parasite [11,111,112]. At the ultrastructural level, the feeder organelle is formed by numerous folds (lamellae) that markedly enlarge the host–parasite contact zone and are fringed by endocytic vesicle-like structures [109]. Freeze fracturing revealed that the membrane folds of the feeder organelle are closed at the attachment site but, on the opposite side, connect with cytoplasmic vesicles in the parasite cytoplasm [113]. The rearrangement of the host cell cytoskeleton induced by invading cryptosporidia appears to lead to the formation of a network for vesicle trafficking, facilitating transport of the nutrients between the host cell and PS [106]. It is assumed that cryptosporidia rely solely on the host for nutrient acquisition and, for this purpose, encode a number of transporters [114]. The presence of ABC-cassette binding proteins at the parasite–host interface supports this hypothesis [112]. It is not clear how cryptosporidia lacking the key *de novo* synthesis of amino acids, fatty acids, and nucleosides take up nutrients directly from their environment (e.g., from the culture medium) at their extracellular stages reported in some in vitro systems. However, as extracellular stages from biofilms have been shown to possess feeder organelles, they may be able to acquire nutrients in a cell-free environment [110,115]. The frequently discussed close affinity and similarities between *Cryptosporidium* and gregarines [8,9,11,45,94,97] raises the question as to whether cryptosporidia obtain nutrients from the host via feeder organelle in a manner analogous to myzocytosis or in another way. The cryptosporidian anterior vacuole (a precursor of the feeder organelle) is indeed strikingly similar in appearance and localisation to the mucronal vacuole of archigregarines and blastogregarines. 

A similar attachment strategy is well documented in certain eimeriids from cold-blooded vertebrates (e.g., *Eimeria* formerly known as *Epieimeria*, *Acroeimeria*, *Choleoeimeria*, some *Goussia*) [116,117,118]. Some authors suggest that the general picture of metabolic interactions between cryptosporidia and host resembles that in *Eimeria* [119], but despite sharing some features with cryptosporidia, the mode of nutrient uptake in epicellular eimeriids may differ. It most likely occurs through the basal PS membrane, enlarged by projections and equipped with abundant pores, thereby increasing the area in contact with the host cell [15,120,121]. Similarly, myzocytosis-based feeding is unlikely in *Eleutheroschizon duboscqi* (Figure 7C–G), as its endogenous stages lack the apical complex and no organelles resembling the flask-shaped structure or mucronal vacuole have been detected in freshly attached *E. duboscqi* parasites [15]. It is not clear whether the complicated attachment apparatus of endogenous *E. duboscqi* stages consisting of lobes and filamentous fascicles organised in rings (Figure 7C,D) is involved in nutrient acquisition. Numerous micropores distributed along the entire parasite pellicle are expected to function in feeding, as they are associated with parasite vesicles and mitochondria [15]. The proposed feeding function applies in particular to micropores distributed at the parasite attachment site. Interestingly, the micropores (except for those at the attachment site) are situated at the bottom of the grooves separating the broad folds forming the parasite surface, as also seen in gregarines. 

The mature stages of *E. duboscqi* tightly enwrapped in a thin PS, forming in a similar way to that in cryptosporidia, are covered by a thick glycocalyx layer, which may hinder the potential fusion of the PS with the parasite surface. Both these apicomplexans, *E. duboscqi* and cryptosporidia, regularly detach (apparently during sample processing) along with their PS from the unmodified compartment of parasitised cell; while cryptosporidia separate in the dense band region, *E. duboscqi* parasites break away from PS at the base, exposing their naked attachment site (=only covered by the parasite pellicle) and leaving the PS inner membrane within the brush border of the host intestine. While the PS of *Cryptosporidium* spp. contains small amounts of filamentous actin [122], the PS of *E. duboscqi* shows increased accumulation of actin filaments that appear be to more stable than those in the surrounding host tissue (Figure 7E). Similarly to cryptosporidia, invading parasites induce the accumulation of host cell F-actin at the base of the PS (Figure 7F) [15]. In addition, the presence of a polymerised form of α-tubulin within the PS (Figure 7G) suggests a role for enterocyte microcilia in the formation of the *E. duboscqi* epicellular niche. However, *E. duboscqi* induces only moderate modifications to the host cell compared to cryptosporidia, which together with host cytoskeleton remodelling leading to microvillous hypertrophy (=elongation and protrusion of host cell microvilli surrounding the attached parasite) also significantly alter the organisation of the HCPM [15,106,113]. The clustering of long and particularly thick host microvilli containing dense F-actin bundles suggests active manipulation of the HCPM by cryptosporidia. Despite sharing a similar attachment strategy with cryptosporidia, *E. duboscqi* has less of a pathological effect on host tissue and, except for a few microcilia that are occasionally attached to the PS surface, does not cause any significant changes (extension) in adjacent microvilli (Figure 7F). Surprisingly, the archigregarine *Ditrypanocystis* also develops within a multimembranous envelope originating from fused enterocyte cilia in the host oligochaete *Enchytraeus albidus* [96]. Host cilia clustering around the attached archigregarine lose microtubular content, resulting in their fusion along with the formation of the PS membrane with a considerably enlarged contact area. The intensively folded ciliary membranes beneath the parasite attachment site give rise to a network of canals, which open in the immediate proximity of the enterocyte apical surface and are in direct contact with the contents of the enterocyte transport vacuoles crossing the HCPM. Neither parasite membrane folds (resembling the cryptosporidian feeder organelle) nor fusion with the HCPM form, and the trilayered pellicle of parasite underlined by subpellicular microtubules is preserved in the contact region [96]. 

The most distinct characteristic shared between cryptosporidia, protococcidia, eimeriids from poikilotherms and gregarines is that they create a highly specialised epicellular niche that perfectly reflects the analogous modes of adaptation for development in similar environments, i.e., morphofunctional convergence [15]. While the extracellular/epicellular apicomplexans are generally of heteropolar nature, intracellular apicomplexan parasites appear to lose their polarity. The majority of epicellular Apicomplexa described above are strictly heteropolar cells exhibiting a high degree of cell polarity with their anterior and posterior ends differing in both function and architecture. All these parasites attach to the host cell/tissue via highly sophisticated apical processes (such as the epimerite/mucron in gregarines, feeder organelle in cryptosporidia and the massive attachment apparatus in *E. duboscqi).* Although epicellular eimeriids, lacking a prominent attachment device, seem to be nonpolar, they create basal projections of the PS equipped with numerous pores resembling the attachment site of *E. duboscqi*. All these parasites (except for gregarines, if one omits the exceptional epicellular niche in *Ditrypanocystis*) are capable of stimulating additional growth, reorganisation and subsequent fusion of host cell microvilli/microcilia along with modifications to the HCPM, resulting in the formation of PS. As a consequence, these parasites develop within the cavity of a protective host-derived envelope separating them from the host internal environment. Unlike vertebrate apicomplexans with intracellular development, the evolutionary selection for these parasites appears to favour the epicellular niche, which allowed them to more effectively avoid a host immune response, at the cost of the parasite becoming fully dependent on its association with the host cell for nutrient uptake [15]. It has been suggested that the formation of a host–parasite interface and attachment devices of epicellular gregarines and cryptosporidia are homologous [11]; however, whether the *Cryptosporidium* feeding organelle represents an intermediary step in the evolution of host–parasite interface between eugregarines and intracellular apicomplexans from vertebrates or a completely innovative novel structure remains to be elucidated. 

### 3.3. Intracellular Parasitism

The cellular tropism of obligate intracellular parasites results from coevolution with their hosts and from the establishment of specific molecular parasite–host cell interactions [123]. Various cell compartments, including the host nucleus, cytoplasm and vacuoles, can serve as shelters for the intracellular parasite, which often depends on the uptake of complex nutrients from the host cell because it cannot synthesise them itself. This coevolution may have been particularly complex for parasites completing their life cycles in successive distinct hosts, such as the malaria parasite that completes its sexual life cycle in *Anopheles* mosquitoes and has two successive asexual developmental phases in hepatocytes and then in erythrocytes. While the molecular exploration of host–parasite interactions within mosquitoes and hepatocytes has been conducted to a lesser extent, current knowledge points to variations in parasite invasive strategies compared to those found in erythrocytic stages, notably different rhoptry and microneme protein repertoires [124]. Ookinetes and sporozoites have tissue- and cell-traversal capacities, before internalisation (for sporozoites) within the PV in liver cells [124]. However, the feeding of intracellular parasites leads to a certain degree of competition between the parasite and the host cell metabolism and even to the erosion of its cytoplasm [125]. As discussed above in the case of epicellular parasites, intracellular protists also modify the host cell structure and metabolism, albeit with a much more severe impact on host tissues. Their pathological effect may lead to final lysis of the parasitised cell, either at the end of the intracellular phase of the parasite’s life cycle or just before its infective stages abandon the host cell to disseminate infection to surrounding cells. In addition, intracellular protists have developed strategies to successfully avoid destruction by host lysosomes, including resisting host cell enzyme attack, escaping from the phagosomal system into the host hyaloplasm, engulfing themselves with lysosome-inhibiting vacuoles and invading host cells lacking the lysosomes [125].

During the invasion of host cells, most intracellular apicomplexans invaginate a portion of the HCPM that eventually seals itself, enclosing the parasite within an intracellular compartment (i.e., within a PV). This process is best described in the human pathogens *Toxoplasma* and *Plasmodium*, which, following internalisation within their respective PV, profoundly modify their host–parasite interface, represented by the PV, in order to sustain nutriment acquisition but also to manipulate the host cell to their benefit. The PVM, which for internalised *Toxoplasma* tachyzoites is mainly derived from the host cell, has a molecular composition that depends on both the invading parasite species and the invaded cell. In *Plasmodium*, the molecular composition of the PVM differs in infected hepatocytes and infected erythrocytes. Moreover, the PVM is closely located to the PPM, and in addition, there may be attachment points between these two membranes as described in *Plasmodium* [126]. Following the establishment of this intracellular niche, the parasite evolves, losing its polarity and extensively modifying the host–parasite interface to allow both nutrient uptake and waste excretion as well as decorating it to build a protective structure against the host defence systems. To this end, the parasite also sends molecules beyond the PVM to manipulate the surface of the host cell (*Plasmodium*) or even the host cell transcriptomic programme (*Toxoplasma*). For example, the malaria parasite extends the PVM in the host erythrocyte cytoplasm by building a structure known as tubulovesicular network and is thought to provide membranes for the formation of Maurer’s clefts, which are responsible for trafficking exported parasite proteins to the surface of the host cell. Notably, a PVM-enclosed intracellular niche is not an absolute necessity for intracellular apicomplexan parasites, as is the case in *Babesia*, where it disappears soon after its formation [127]. Apicomplexan parasites of vertebrates must acquire essential nutrients from their host cells, ranging from vitamins and cofactors to glucose, amino acids, purines and certain lipids, to name but the main categories, and a close examination of the nutrient acquisition routes illustrates the complexity and specificity of each situation. The malaria transportome (the name given to its transporter systems, comprising 19 channels, 96 carriers and 29 pumps) currently represents ~2.5% of its predicted proteome, which is a low percentage compared to many other reference (metazoan) genomes but similar compared to other apicomplexan parasites (~2 to 2.5% in *Cryptosporidium* and *Theileria* for example) [83]. Global transportome expression depends on the developmental stage, reflecting different parasite needs according to the particular niches they occupy in their hosts or vectors [83]. It has been established that the various parasite membrane systems (PPM, organellar membranes, etc.) are equipped with different sets of transporters, depending on their specific biochemical properties and physiological roles in nutriment uptake, waste extrusion or electrochemical gradient maintenance for example. Functional studies have revealed that there is little redundancy in the *Plasmodium* transportome; about two-thirds of the genes appear to be essential for at least the erythrocytic stages [83]. This ‘minimalist’ transportome has been interpreted to reflect the relatively stable and nutrient-rich environment elected by the malaria parasite, which allowed a streamlining of the transporters repertoire [83]. Whether the basal apicomplexan parasites will also reveal streamlined transportome repertoires will be interesting to determine. Importantly, intracellular parasites may also rely to some extent on host-cell machinery for nutrient uptake. For example, glucose uptake to the benefit of the *Plasmodium* parasite within erythrocytes involves mainly uptake via the host glucose transporter glu1 at the erythrocyte surface and then parasite-specific transporters at the level of the PPM [128]. Intracellular parasites also acquire nutrients via endocytic-like pathways. The best-described route is that of haemoglobin uptake by *Plasmodium* in its erythrocytic stages, involving a cytostome at the PVM/PPM interface, which produces vesicles that are then trafficked towards a lysosome-like derived organelle called the digestive vacuole (or food vacuole) where the host haemoglobin is broken down by a cascade of proteolytic enzymes into smaller peptides [129,130]. The molecular components of this endocytic pathway are beginning to be identified [131].

**Figure 8:**
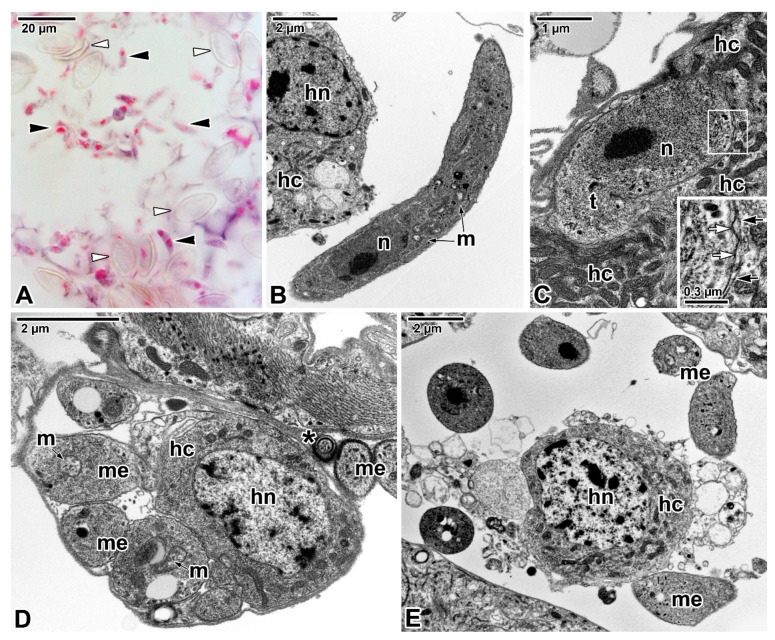
Neogregarine *Mattesia dispora* parasitising the fat body of a larval Mediterranean flour moth (*Ephestia kuehniella*). (**A**) Sectioned body of larval host showing the absolute destruction of its fat body, which was replaced by autoinfective oocysts (already empty) and sporozoites released from them (LM—histological section stained with haematoxylin and Best’s carmine). (**B**) Free sporozoite (TEM). (**C**) Hypodermal cell newly invaded by a macronuclear merozoite (transforming into a trophozoite stage enclosed within a parasitophorous vacuole); the insert shows a detail of the host–parasite interface marked by a white rectangle (TEM). (**D**) Intracellular merozoites (TEM). (**E**) Merozoites emerging from a degenerated and vacuolated host cell (TEM). *asterisk*—cross-sectioned conoid and polar rings of a free merozoite, *black arrow*—parasitophorous vacuole, *black arrowhead*—free sporozoites, *hc*—host cell, *hn*—host cell nucleus, *m*—parasite mitochondria, *me*—merozoite, *n*—parasite nucleus, *t*—trophozoite, *white arrow*—parasite plasma membrane, *white arrowhead*—oocysts.

However, intracellular development appears to be a less preferred strategy for parasitism in apicomplexan parasites of invertebrates. Although a few studies have been conducted on the intracellular niche of these parasites, they are mostly descriptive works based solely on ultrastructural observations. An intracellular phase has been reported in the life cycle of some neogregarines. In contrast to other gregarines, neogregarines (mostly invading insect fat bodies, haemocoel, Malpighian ducts and intestines) are considered potential candidates for biological control of insect pests as their hosts often fail to survive heavy infection [132]. The development of the neogregarines starts in the host intestine, with excysted sporozoites migrating through the intestinal epithelium into the haemocoel and reaching the target organ/tissue. Their vegetative stages are usually intracellular or free-lying within tissues, with no or only a reduced attachment region [133]. Some neogregarines have been documented to develop within a PV [134,135,136], which most likely forms as the consequence of an interaction between the parasite invasion and host defence, as reported in *Galleria mellonella* [136] and *Ephestia kuehniella* parasitised by *Mattesia dispora* (Figure 8C). 

A different parasitism strategy has been reported for *Farinocystis tribolii*, a neogregarine parasitising the fat body of *Tribolium castaneum*, where the only response to infection observed was the accumulation of host mitochondria around the meronts and other nonmotile parasite stages [132], putatively to scavenge host metabolites; a similar reaction has been documented in ultrathin sections of *E. kuehniella* hypodermal cells parasitised by *M. dispora* (Figure 8C). No melanisation or phagocytosis has been documented in host insects parasitised by these neogregarines [132].

Intracellular development appears to limit the growth of neogregarine trophozoites (as opposed to the huge trophozoites in epicellular gregarines), which, due to asexual multiple divisions, called merogony, produce a generation of merozoites that attack other host cells/tissues and spread the infection [47]. In addition, some of these insect pathogens also produce autoinfective oocysts (sexual phase), contributing to the rapid spread of infection and resulting in severe parasitisation (Figure 8A,B) [132,137,138,139,140]. Neogregarines are frequent causes of insect morbidity and mortality due the significant pathological changes they cause in their hosts, including host cell lysis and destruction of tissue invaded by the merogony stages [133,138,141]. The nutritional requirements of neogregarines are often very high, and during their development, the host tissues are gradually destroyed and replaced by the parasite’s oocysts (Figure 8A) [139]. The best-studied neogregarine with respect to the host pathology is *M. dispora*, which is frequently found to parasitise the fat body of the Mediterranean flour moth (*E. kuehniella*). The vacuolation and degeneration of the host cells infected by *M. dispora* have been reported in ultrastructural studies (Figure 8D,E) [138]. Another study reported that *M. dispora* parasitises a host cell with no apparent response, but its meront stages reduce the excretion and deposition of fat within the parasitised cell through an unknown mechanism, resulting in overgrowth of the fat body by cytoplasm-rich cells lacking the lipid vacuoles [139]. As the infection progresses, the affected host cells become hypertrophied with the nucleus clearly pushed back. Reduced host activity and food intake are often observed during micronuclear merogony, whereas during macronuclear merogony (when the infection spreads to the haemocoel and hypoderm) the larvae are lethargic with markedly reduced movement. Heavily parasitised larvae turn pale and stop eating, and the infection spreads throughout their body, resulting in the destruction of their tissues that are replaced by neogregarine oocysts floating freely in the haemocoel (Figure 8A); some of these oocysts are autoinfective and release their sporozoites (Figure 8A,B). Before the death of these larvae, a change in the colour of their body to an intense pink was documented [138]. Complete destruction of the fat body (tissue that cannot regenerate and is needed for metamorphosis in insects), its replacement by the parasite and significant pathology in the host insect (usually with fatal consequences) has also been reported in *T. castaneum* parasitised by *F. tribolii* and larval *Aeshna grandis* and *Libellula quadrimaculata* parasitised by *Syncystis aeshnae* [132,135]. These observations indicate that these neogregarines multiply within the host body until it is completely consumed and filled by the parasite oocysts. Neogregarines accumulate in their hosts, in contrast to less pathogenic eugregarines, whose gametocysts are continuously expelled from the host body [132]. Of particular interest is the parasitism strategy of *Ophryocystis* neogregarines, with an intracellular form of development in lepidopteran hosts (*O. elektroscirrha* developing in the hypodermis of the monarch butterfly, which is only rarely fatal in natural populations of its host) but with an extracellular form in coleopteran hosts (*O. schneideri* and *O. mesnili*) [47]. The extracellular development of *Ophryocystis* neogregarines also includes merogony with the production of two types of meronts—micronuclear conical or mycetoid forms and a macronuclear vermiform form that transforms into rounded gamonts. The micronuclear meronts are in fact epicellular parasites attached by pseudopods (= rhizoids) to ciliated cells of Malpighian tubules in tenebrionid beetles. These pseudopods are packed with cytoskeletal elements (parallel fibrils and microtubules) and interdigitate with microvilli of the brush border in Malpighian tubules [47,142] in a unique manner resembling the aforementioned strategy of attachment in epicellular eimeriids in cold-blooded hosts. The fibres, which run in all directions in the meront cytoplasm, organise themselves in parallel within rhizoids and form ‘swellings’ in some areas. The parasitism strategy (epicellular vs. intracellular) in *Ophryocystis* suggests that the parasite has specifically adapted to the internal environment of a particular host type (Lepidoptera vs. Coleoptera). 

Almost nothing is known about nutrient uptake in neogregarines. There is only sketchy information from a limited number of neogregarine representatives that could indicate how these little-studied basal apicomplexans could receive nutrients from their hosts. A structure resembling the mucron-like organelles in eugregarines has been documented in *M. grandis* [134]. It consists of the specialised zone of parasite plasmalemma from which the fine fibrillar structures emerge and extend in a large vacuole with abundant anastomosing protrusions (suggesting its nutritional function) and with its outlet surrounded by former apical polar rings. However, unlike the apical complex (formed by the conoid, polar ring, rhoptries, micronemes and subpellicular microtubules) present in a major portion of the life cycle of neogregarines studied at the ultrastructural level (including *M. grandis*, *M. dispora*, *F. tribolii* and *S. aeshnae*) [134,135,138,143,144,145], the presence of mucron-like organelle, most likely originating from the apical complex, appears to be restricted to younger meronts (with 1–4 nuclei) of *M. grandis*. In contrast, merozoites of *M. grandis* are equipped with a well-developed apical complex connected to a large vacuole as observed in the typical mucron of archigregarines and blastogregarines. In *M. grandis* and *F. tribolii*, the presence of micropores (pinocytotic vesicles or similar invaginations of the parasite pellicle) that could serve for parasite feeding has been reported only in gamonts, while the other parasite stages appear to lack these structures [132,134,146]. In contrast, typical micropores interrupt the pellicle of *S. aeshnae* merozoites [135]. Mitochondria (usually with vesicular cristae) have been observed in various developmental stages of neogregarines, being most abundant in their gamont stages [134,135,138,143,144,145]. The presence of amylopectin granules (in lower numbers compared to eugregarines) and lipid droplets has been also reported in the above-mentioned species. The differences in feeding equipment between the individual developmental stages of the neogregarines studied so far may be due to insufficient data analysis or may simply suggest that neogregarines take up nutrients in different ways during their life cycle. These feeding modes could comprise the myzocytosis-based feeding in merozoites (and probably sporozoites) equipped with apical complex connected to a ‘mucronal’ vacuole, a suction process (similar to that described for *Lecudina* eugregarines) in trophozoites and young meronts of *M. grandis* with mucron-like organelle and endocytotic uptake via micropore(s) in older (gamont) stages and probably free zoites.

Transient intracellular stages have been also reported during the development of some archigregarines, where the released invasive sporozoites migrate from the host intestinal lumen through the epithelium and reach the basal lamina where they develop into trophozoites entering the merogony. However, these findings are rare, and no detailed description of the life cycle of these parasites has been published to date. One example of the presence of an intracellular phase in the archigregarine life cycle is the discovery of intraepithelial cysts located near the basal lamina and containing several dozen merozoites in polychaetes highly parasitised by *S. hollandei* [133]. Another example is the occurrence of small intracellular trophozoites of *S. pygospionis* (with a subcellular organisation identical to epicellular well-developed trophozoites) contained in a PV [14]. The cytoplasm of host enterocytes surrounding the PV with parasites is rich in mitochondria, endoplasmic reticulum, vesicles and dense fibrillar material, whereas the rest of the host cytoplasm is lucent with few organelles. Although the host cell apparently responds to the presence of intracellular stages of archigregarines, it is not known whether these stages receive food or survive on energy resources mostly in the form of amylopectin granules.

Probably least known are the nutritional strategies of coccidian-like apicomplexans with a poorly understood developmental cycle, such as agamococcidia. These enigmatic parasites, with no morphological evidence of sexual reproduction, seem to lack gamont stages as well as merogony [147]. Their life cycle is described as streamlined where sporozoites (penetrating the host intestine and persisting in the connective tissue, gonads and coelom) develop into trophozoites, which later form numerous sporoblasts by superficial budding [148]. Several recent papers reported observations of *Rhytidocystis* spp. from polychaete hosts, where their relatively large trophozoites (apparently immotile) develop intratissularly and/or intracellularly, lacking the PV (except for putative sporozoites in *R. polygordiae*) [147]. The typical apicomplexan pellicle covering these parasites is organised in longitudinal series of small transverse folds with numerous micropores continuous with the IMC [147]. The trophozoites lack organelles of apical complex and attachment structures, and their cytoplasm is packed with reserve amylopectin granules, vacuoles, lipid droplets, Golgi apparatus, cisternae of the endoplasmic reticulum, dictyosomes and abundant giant mitochondria with tubular cristae localised at the parasite periphery just beneath the pellicle [149]. The absence of apical complex in trophozoites suggests that they feed via micropores through endocytosis [147], or by importing soluble metabolites through specific transporters, or use storage macromolecules.

## 4. The Storage Polysaccharide in Apicomplexan Parasites

Amylopectin (termed paraglycogen in some studies) is known to accumulate in Apicomplexa during their vegetative developmental stage and is assumed to represent the energy reserve required for (i) survival of their exogenous oocyst stage, (ii) excystation process, (iii) invasion of the host cell and, finally, (iv) the transition from one developmental stage to another [47,150,151,152]. In gregarines, these storage polysaccharides occur in the form of spherical or ovoid bodies (>1 μm) (Figure 4B), which are insoluble in cold water but soluble in water at 45–60 °C, and are very sensitive to saliva [47]. These granules stain brown with iodine (characteristic of glycogen); however, a dark cross phenomenon may be observed when they are exposed to polarised light, suggesting their starchy nature and thereby leading to confusion in terminology—paraglycogen, glycogen and zooamylon. Enzymatic assays performed on *S. hollandei* and *L. tuzetae* showed the centre of the granules to be more fragile than the periphery with thin filaments organised concentrically and radially [47]. Biochemical and digestion analyses of these granules performed in *G. blaberae* revealed that they consist of a homopolysaccharide composed of chains of α-1,4-linked glucose molecules linked by α-1,6-glucosidic interchains (with an average length of 19 glucose molecules) and that the unit chain profile of *G. blaberae* amylopectin, intermediate between glycogen and plant amylopectin, is in fact similar to that of sweet corn phytoglycogen [153]. In conclusion, the *G. blaberae* storage granules are of amylopectin nature with an average chain length of about 20 glucose molecules and with properties similar to those of *Eimeria* coccidia [154]. It has been experimentally demonstrated that *E. tenella* sporozoites consume their cytoplasmic amylopectin when incubated in aerobic conditions without substrate enrichment, while the consumption of amylopectin was reduced by the addition of glucose, fructose, mannose and maltose [155]. This in vitro study showed that sporozoites take the glucose from the incubation medium and catabolise it into CO_2_ under aerobic conditions. Interestingly, during *E. tenella* sporulation, the amylopectin granules are degraded by an amylopectin phosphorylase; while the number of granules decreases, the shape and size of the granules do not change, suggesting that the enzymatic attack first strikes and completely degrades one granule before moving on to the next one [152]. Unsporulated oocysts of *E. tenella* contain large amounts of carbohydrates, including amylopectin, mannitol and glucose. Mannitol accumulation during the early stages of sporogony, accompanied by a rapid decrease in amylopectin and free glucose, suggests that the glucose released from amylopectin could be involved in mannitol synthesis [156]. Tachyzoites of *T. gondii* growing in acidic medium produce a large quantity of amylopectin, suggesting that amylopectin synthesis is a metabolic adaptation to environmental fluctuations [150]. The *T. gondii* amylopectin*,* composed of α-1,4-linked glucan linear chains with a small proportion of α-1,6 branches [150], is similar to the semicrystalline floridean starch accumulated by red algae [157]. It has been suggested that the preservation of this inherited pathway may be required for apicomplexans harbouring the dormant cyst stages in their life cycles, while in parasites such as *Plasmodium* that propagate with no dormant cysts, the storage polysaccharide metabolism was lost [157]. The synthesis and accumulation of amylopectin granules in the cytoplasm together with the acquisition of the apicoplast can be considered as tracers of the evolutionary origin of Apicomplexa to red algae [150]. The cytoplasmic storage polysaccharides in apicomplexan parasites (e.g., *T. gondii* and *C. parvum*), dinoflagellate *Crypthecodinium* and red alga *Cyanidioschyzon merolae* are synthesised via a UDP-glucose-based metabolic pathway, which is similar to the glycogen pathway in fungi and animals but distinct from the plant starch pathway [157].

## 5. Conclusions and Future Perspectives

**Figure 9:**
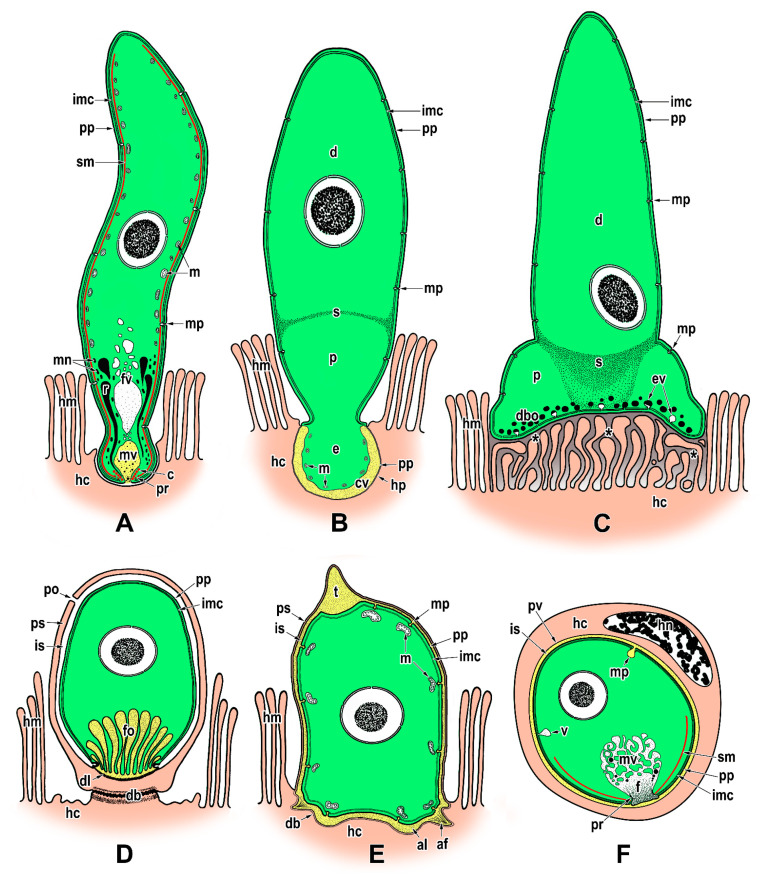
Schematic representation of host–parasite interactions in basal apicomplexans. (**A**) Blastogregarine or archigregarine trophozoite developing epicellularly, attached to the luminal side of the host epithelial cell by a well-developed mucron. See also Figure 1A,B, Figure 2A–E, and Figure 6A–C. (**B**) Eugregarine trophozoite developing epicellularly, anchored to the luminal side of the host epithelial cell by a simple epimerite. See also Figure 3A,B, Figure 4A–C and Figure 6D. (**C**) Eugregarine gamont developing epicellularly, attached to the host microvilli by a modified (sucker-like) protomerite. See also Figure 5B. (**D**) Cryptosporidian trophozoite developing epicellularly within a parasitophorous sac of host cell origin. See also Figure 7A,B. (**E**) Protococcidian trophozoite developing epicellularly within a parasitophorous sac of host cell origin. See also Figure 7C–G. (**F**) Neogregarine trophozoite developing intracellularly within a parasitophorous vacuole. See also Figure 8C. The diagrams are based on our personal observations enriched by published data. Three colours are used to distinguish between the parasite (in green); the host cell, including its parts modified due to parasitisation (in pink); and the contact zone between the host and the parasite (in yellow), where the interrelationships of the two organisms become more intimate. In the case of host–parasite cellular interactions in protococcidia (**E**) and neogregarines (**F**), the internal space between the parasite and PS/PV may serve as a transitional zone for intensive interactions between the host and its parasite. The fragmented vacuoles in blastogregarines/archigregarines (**A**) and the mucronal vacuole in neogregarines (**F**) remain colourless but are thought to be involved in parasite feeding. Emphasis is given only on the characteristic organisation of selected structures/organelles, inclusions and organelles randomly dispersed in the cytoplasm are not shown for better clarity of the schematic drawing. *af*—attachment fascicle of filaments, *al*—attachment lobe, *asterisks*—space between the intestinal epithelium and the attached protomerite filled with crumpled host microvilli deposited in a dense adhesive material, *c*—conoid, *cv*—cortical vesicle, *d*—deutomerite, *db*—dense band, *dbo*—dense bodies, *dl*—dense line separating the feeder organelle from the filamentous projection of the PS, *e*—epimerite, *ev*—exocytotic vesicles (at various stages of emptying their contents) directly linked to the pores interrupting the IMC covering the protomerite apical region, *f*—mucronal fibres, *fo*—feeder organelle with membranous lamellae, *fv*—fragmentation of the mucronal vacuole, *hc*—host cell, *hn*—host cell nucleus, *hm*—host microvilli, *hp*—host cell plasma membrane, *imc*—inner membrane complex, *is*—internal space between the parasite and PS/PV, *m*—parasite mitochondria, *mn*—micronemes, *mp*—micropore, *mv*—mucronal vacuole (in neogregarines with anastomosing protrusions and rhoptries/their remnants), *p*—protomerite, *pp*—parasite plasma membrane, *po*—pore (or incomplete fusion) on the PS, *pr*—polar ring(s), *ps*—parasitophorous sac, *pv*—parasitophorous vacuole, *r*—rhoptry, *s*—fibrillar septum separating the protomerite from the deutomerite, *sm*—subpellicular microtubules, *t*—tail of the PS, *v*—pinocytotic vesicle.

This review underlines the huge diversity of subcellular organisation and highly specialised adaptations to a parasitic lifestyle in basal lineages of Apicomplexa, which are perfectly reflected in their various attachment and feeding strategies. Here we summarised the current state of knowledge on the attachment and nutrient uptake strategies displayed by these parasites, focusing on their trophozoite stages (Figure 9A–F). Current studies clearly indicate the need for further research on basal lineages of Apicomplexa, especially from the marine environment, to establish a more realistic phylogenetic framework of Apicomplexa and to identify primitive and advanced parasitism strategies and describe their emergence and evolution. Another crucial question is whether these specific modes of parasitism originated once or independently several times. To fully understand the origin and evolution of the nutritional mechanisms in Apicomplexa, it is first necessary to tackle many questions, among which we emphasise the following: (i) Which of the molecular components constituting the apical complex in vertebrate parasites also compose the apical complex in parasites of invertebrates and which are missing? Does this inventory explain why vertebrate apicomplexans appear to prefer intracellular niches while most invertebrate parasites do not? Do invertebrate apicomplexans have additional actors that may shed light on their different functions? (ii) Do archigregarines and blastogregarines feed exclusively by myzocytosis, or do they also obtain nutrients from their environment through osmotrophy and/or cell surface-mediated endocytosis? (iii) Have eugregarines shifted from myzocytosis-based acquisition of nutrients to more derived ‘vacuole-mediated’ nutrient acquisition by developing extremely diverse attachment devices dedicated to both attachment and feeding? (iv) To what extent are these structures comparable to the PV of intracellular vertebrate parasites? (v) Does the *Cryptosporidium* feeding organelle represent an intermediary step in the evolution of the host–parasite interface between eugregarines and intracellular apicomplexans such as *Toxoplasma* and *Plasmodium*, or is it a completely new innovative structure? (vi) To what extent are the endocytic pathways used by *Plasmodium* and *Toxoplasma* parasites to acquire nutrients comparable to the endocytic modes used by basal apicomplexan representatives? All these and many other unresolved issues, particularly those concerning the (until recently almost ignored) basal lineages form a barrier that prevents us from truly understanding the origin and evolution of the parasitic strategy in Apicomplexa.

## Data Availability

No new data were created or analysed in this study. Data sharing is not applicable to this article.

## Abbreviations

AMA-1	Apical membrane antigen 1
CLSM	Confocal laser scanning microscopy
FE	Freeze-etching
FITC	Fluorescein isothiocyanate
GRA	Dense granule protein
HCPM	Host cell plasma membrane
IFA	Indirect immunofluorescent assay
IMC	Inner membrane complex
LM	Light microscopy
PPM	Parasite plasma membrane
PS	Parasitophorous sac
PV	Parasitophorous vacuole
PVM	Parasitophorous vacuole membrane
RON	Rhoptry neck protein
ROP	Rhoptry (bulb) protein
SEM	Scanning electron microscopy
TEM	Transmission electron microscopy
TRITC	Tetramethylrhodamine isothiocyanate
